# Abstracts from Foreign Journals

**Published:** 1902-12

**Authors:** 


### ABSTRACTS FROM FOREIGN JOURNALS.

### FRENCH.

UNDER THE DIRECTION OF S. J. J. HARGER, V.M.D.,
PHILADELPHIA, PA.

TRANSMISSION OF TUBERCULOSIS TO MAN BY ACCIDENTAL INOCULATION AND EXPERIMENTAL REINOCULATION OF A CALF. In May, 1900, Overbeek, of Utrecht, in making a post-mortem on a cow affected with generalized tuberculosis, accidentally cut the small finger of the right hand of one of the flayers in the abattoir. In February, 1902, this man applied for treatment at the surgical clinic of the Faculty de Médecine at Utrecht. The small wound healed in three days after the accident, but the finger remained painful and gradually became swollen.

Examination showed the skin to be thickened and of a bluish color, especially at the level of the articulation of the last two phlanges, fissured over the centre of the swelling, covered with small scabs, and painful to pressure. The cubital ganglion of that side had become as large as a pea. During the last year the man had coughed, expectorated, and at the summit of the right lung dulness and moist râles were detected; the expectoration contained no bacilli.

Three guinea-pigs were inoculated into the inside of the thigh by introducing subcutaneously two pieces of skin obtained from the finger, two died from tetanus, and the third showed generalized tuberculosis seventy days after the inoculation. Two other guinea-pigs inoculated with the pulp of the tubercular spleen obtained from the latter were found affected with tuberculosis when killed. The result was identical in guinea-pigs inoculated with pieces of the cubital ganglion. Tubercle bacilli in large numbers were found in these lesions.

The lesions of the skin and cubital ganglion were, therefore, without doubt, tubercular. The microscope revealed in the skin tubercles, giant cells, and in the ganglion caseation; bacilli were not numerous—in strong contrast with the lesions in the guinea-pigs.

On May 15th a heifer, three months old, was inoculated under the skin of the neck with 2 c.c. of an emulsion in sterilized normal salt solution of the splenic pulp of the guinea-pig inoculated with a piece of the man's skin. There were swelling at the seat of inoculation, tumefaction of the cervical ganglia of that (right) side, diarrhœa, cough from time to time; animal was destroyed July 12th.

*Necropsy.* Tubercular granuloma as large as a fist at the seat of inoculation, containing a cavity filled with soft, caseous material; cervical ganglia hypertrophied, 12 cm. long, and 6 cm. wide, calcified at points, and showing disseminated, caseous centres; numerous tubercles upon the visceral pleura and in the lungs, with caseous pneumonia centres; bronchial and mediastinal glands hypertrophied and tubercular, and tubercles in the liver and spleen. Microscopic examination revealed numerous bacilli in these lesions.

Guinea-pigs inoculated with the product of these lesions died from tuberculosis.

According to Sprongk and Haefnagel, the tubercle bacilli of man and of the bovine are only two races of the same species; that the bacillus of tuberculosis in man can acquire the properties of the bacillus of bovine tuberculosis by several passages through bovine animals; that inversely the bacillus of bovine tuberculosis, by passing through man, may become identical with the bacillus of human tuberculosis.

This observation leaves no doubt upon the possible infection of man by tubercular bovines through milk, butter, and meat.—*Semaine Médicale.*

Botryomycosis in the Mammary Gland of a Mare. Subject, a mare, twelve years old, and emaciated, observed by Dr. Unterhoessel, of Bologne. The mammary tumor extended downward to the hock and forward to the umbilicus. It was hard, nodulated on its surface, the nodules varying in size from that of a hazelnut to that of a walnut, and some of them discharged a yellowish-brown, grumous pus.

*Necropsy.* The glandular tissue had almost completely disappeared from the pressure of the parasitic neoformation, which weighed 35 kilos. Macroscopic, microscopic, and bacteriological examination showed the tumor to be botryomycosis. No tumors were found in any other part of the body—further evidence that botryomycosis has no tendency to metastasis. This growth was identical with some cases of so-called "scirrhus cord," which at times presents identical characters.—*Berliner Thier Wochenschrift.*

Pathological Importance of the Larvæ of the Gastrophilus in the Stomach of the Horse. Perroncito has studied the lesions of this parasite, commonly called "bots."

These larvæ, when quite small, firmly fasten themselves to the epithelium of the mucous membrane by their perforating mandibles and by two mandibular hooklets that develop subsequently. They penetrate ordinarily as far as the cephalic ring, desquamate the epithelium, and cause the formation of a cavity with a raised and convex border and a cicatricial bottom. They may penetrate deeper, making a cavity from ½ to 7 mm. deep, whose bottom is the submucosa, which is the seat of an inflammatory process; this alteration may even involve the entire stomach wall. The irritating action of the mandibular hooklets and the bristles covering them may extend over a more or less larger area. When the first layer of the muscular tunic is affected the connective tissue proliferates and the gastric wall becomes thickened; the cicatricial tissue compresses the muscular fibres and causes them to atrophy and disappear. In some places this process may extend to the external muscular tunica; the bottom of the ulcer is converted into cicatricial tissue, and the peritoneum at this point thickened; the wall of the stomach may present patches of calcareous infiltration.

Horses infected with the larvæ of the gastrophilus are more susceptible to infectious diseases because pathogenic microbes can more readily penetrate through the ulcerations of the mucous membrane. Under such conditions influenza, pneumonia, and anthrax are more fatal when the probable channel of infection is the solution of continuity of the wall of the stomach.

Flohil has seen a colt suffering from several attacks of colic with fever, which finally succumbed to septicæmia. He attributed the infection to solutions of continuity by the larvæ of the œstrus in the wall of the œsophagus.

Numan has seen a case of perforation of the stomach by the gastrophilus, and Conti a colt destroyed by rupture of the organ; the stomach contained many larvæ. The gastric wall becomes further weakened by the action of micro-organisms producing suppuration and gangrene.—*Rev. Vet.*

Treatment of Parturient Paresis. Without deprecating the good results obtained from Schmidt's treatment, de Jong, considering that this treatment often fails and that death is caused by cardiac paralysis of a toxic nature, has substituted for iodide of potash a solution of salicylate of sodium and caffein. This solution is rapidly absorbed, and counteracts the depressing effects of the toxin. "The treatment has given surprising results."—*Ann. de Med. Vet.*

